# Intersectional inequalities in the transition to grandparenthood and cognitive functioning: A longitudinal Multilevel Analysis of Individual Heterogeneity and Discriminatory Accuracy (MAIHDA)

**DOI:** 10.21203/rs.3.rs-3248051/v1

**Published:** 2023-08-10

**Authors:** Enrique Alonso-Perez, Jan Paul Heisig, Michaela Kreyenfeld, Paul Gellert, Julie Lorraine O’Sullivan

**Affiliations:** Charité – Universitätsmedizin Berlin, corporate member of Freie Universität Berlin, Humboldt-Universität zu Berlin, and Berlin Institute of Health, Institute of Medical Sociology and Rehabilitation Science, Charité – Universitätsmedizin Berlin, Berlin, Germany; Research Group “Health and Social Inequality”, WZB Berlin Social Science Center, Berlin, Germany / Institute of Sociology, Freie Universität Berlin, Berlin, Germany; Social Policy Groups, Hertie School, Berlin, Germany; Charité – Universitätsmedizin Berlin, corporate member of Freie Universität Berlin, Humboldt-Universität zu Berlin; Berlin Institute of Health, Institute of Medical Sociology and Rehabilitation Science, Charité – Universitätsmedizin Berlin, Berlin, Germany; Charité – Universitätsmedizin Berlin, corporate member of Freie Universität Berlin, Humboldt-Universität zu Berlin; Berlin Institute of Health, Institute of Medical Sociology and Rehabilitation Science, Charité – Universitätsmedizin Berlin, Berlin, Germany

**Keywords:** intersectionality, healthy aging, cognition, grandparents, intergenerational ties

## Abstract

**Objectives:**

With aging societies, more people become vulnerable to experiencing cognitive decline. While normal aging is associated with a deterioration in certain cognitive abilities, little is known about how social determinants intersect to create late-life cognitive functioning inequalities. Simultaneously, the role of grandparenthood is central for older adults and their families. There are indications that social determinants intersect to modulate the effect of the transition to grandparenthood, but further evidence is needed. Our study investigates the relation of transition to grandparenthood with cognitive functioning and explores differences across intersectional strata.

**Methods:**

Using longitudinal data from the Survey of Health, Ageing and Retirement in Europe, we analyzed a sample of 19,953 individuals aged 50–85 without grandchildren at the baseline. We applied Multilevel Analysis of Individual Heterogeneity and Discriminatory Accuracy to investigate cognitive functioning differences across 48 intersectional strata, defined by sex/gender, migration, education, and occupation. We allowed the impact of becoming a grandparent to vary across strata by including random slopes.

**Results:**

Intersectional strata accounted for 17.43% of the overall variance in cognitive functioning, with most of the stratum-level variation explained by additive effects of the stratum-defining characteristics. Transition to grandparenthood was associated with higher cognitive functioning, with a stronger effect for women. Stratum-level variation in the grandparenthood effect was modest.

**Discussion:**

This study highlights the importance of social determinants for understanding heterogeneities in the association of transition to grandparenthood with cognitive functioning. Adopting an intersectional lens is useful to decompose inequalities and derive tailored interventions to promote equal healthy aging.

## Introduction

Growing longevity and extended lives entail an increased proportion of people at risk of late-life cognitive decline (United Nations, 2015). While cognitive functioning has a genetic component, cognitive differences are also strongly affected by environmental exposures accumulated throughout the life course (Deary et al., 2022). Social determinants such as sex/gender, race/ethnicity and socioeconomic status (SES) combine to shape cognitive functioning, especially at their intersections (Forrester et al., 2019).

Recent demographic changes have raised interest about the health and well-being of older generations and related intergenerational processes (Chang et al., 2019). The transition to grandparenthood is an increasingly common and important event in the life course of older adults and their families (Skopek, 2021). This transition has shown associations with health and well-being, although the direction of the effect remains unclear: Becoming a grandparent can generate psychological benefits through social interaction or positive emotions (Taubman – Ben-Ari et al., 2018), but also threaten mental health due to increased stress or by raising the awareness of feeling older (Tanskanen et al., 2019). The nature and strength of the association between the transition to grandparenthood and healthy aging is stratified by social position, understood as the intersection of multiple social determinants that shape lived experiences (Dolbin-MacNab & Few-Demo, 2018). While evidence suggests that the mental health benefits of grandparenthood are stronger for individuals in more privileged social positions (Di Gessa et al., 2022), investigations of the moderating role of social position in such relation remain scarce (Sheppard & Monden, 2019).

Despite the growing interest in how dimensions of social stratification are intertwined, practically no quantitative research has investigated cognitive functioning from an intersectional perspective (Hale et al., 2022). In the present study we investigate inequalities in healthy aging by studying the association of the transition to grandparenthood with cognitive functioning, and how the impact differs across intersectional strata defined by sex/gender, migration, education, and occupation.

## Background

### Healthy aging and cognitive functioning

Cognitive functioning, understood as the process and the abilities for acquiring and processing information, reasoning and decision-making, is a key component of daily functioning (Jeon et al., 2022). Cognitive decline can negatively impact an individual’s ability to carry out everyday tasks, which implies a loss of independence and quality of life (Thorvaldsson et al., 2016). Lower levels of cognitive functioning are closely linked to deteriorated mental health and well-being, making it a fundamental part of healthy aging (Martinussen et al., 2019).

While normal aging is associated with a decline in cognitive functioning due to natural brain changes, social disparities in cognitive functioning cannot be explained by these biological processes alone (Bishop et al., 2010). Higher mental complexity of the main lifetime occupation is predictive of the level and trajectory of change in cognitive functioning (Finkel et al., 2009). Likewise, the cognitive enrichment theory posits that intellectual and social activities improve cognition, meaning that inactive lifestyles can accelerate decline in cognitive functioning, whereas exposure to various stimuli can prevent or delay decline in cognitive functioning (Hertzog et al., 2008). Further, there is evidence regarding substantial cognitive inequalities among diverse socio-environmental contexts for older adults (Forrester et al., 2019). Notably, sex/gender and SES are key determinants of disparities in trajectories of cognitive functioning (Walsemann et al., 2022).

### Transition to grandparenthood and healthy aging

As the intergenerational overlap is being prolonged due to increasing life expectancy, research has expanded to encompass the dynamics of grandparent-grandchildren relations (Taubman – Ben-Ari et al., 2018). These relationships can be vital sources of support and social integration within families, but also of stress and additional burden in late-life (Bordone et al., 2023). At the same time, growing residential mobility could diminish the benefits of becoming a grandparent, as grandchildren may live further away (Hank et al., 2018).

The transition to grandparenthood can increase social interaction, help maintain positive emotions like sense of purpose and belonging, as well as strengthen intergenerational ties (Arpino & Bordone, 2014). On the other hand, negative self-perceptions such as feeling older, increased stress or burdens related to care provision and intergenerational re-allocation of care resources are linked to mental health decline in older adults (Bordone et al., 2023; Tanskanen et al., 2019). A growing body of literature is exploring grandparenthood and its consequences for older adults’ mental health and well-being, both regarding the event of transition to grandparenthood itself and the practices that follow in the new status, namely behavioral aspects and grandparenting (e.g., childcare). As Bordone et al. (2023) propose, it is appropriate to differentiate both aspects. Focusing on the transition, despite early calls to explore the impact of such an important life-course event (Cunningham-Burley, 1986), the scarce research that explored its effect on healthy aging mostly involved cross-sectional analyses (i.e., comparing grandparents with non-grandparents) that generated ambiguous findings. Hence, looking at the transition longitudinally is needed to better understand its consequences in healthy aging.

The few studies investigating the transition to grandparenthood with large-scale European data provide modest evidence of benefits for mental health and well-being (Bordone & Arpino, 2019; Di Gessa et al., 2020; Leimer & van Ewijk, 2022; Sheppard & Monden, 2019; Tanskanen et al., 2019). In line with cross-sectional evidence (Arpino & Bordone, 2014), these studies show effect heterogeneities based on social determinants, such as more pronounced benefits for women. While such transition is a chance to promote healthy aging for older adults (Taubman – Ben-Ari et al., 2018), evidence remains scarce and inconclusive, hence further research is needed regarding how social position influences the relation between transition to grandparenthood and cognitive functioning.

While several studies highlight the importance of social determinants for late-life cognitive functioning, most have analyzed social determinants separately rather than in combination (Cicero et al. (2023) for an exception). This leads to an incomplete understanding of the complex ways in which life-course cumulative exposures and critical events may impact cognitive functioning. Considering multiple axes of interaction between social determinants can help us address critical knowledge gaps related to cognitive functioning disparities in older adults (Hale et al., 2022). Since intersectionality has rarely been applied to explore healthy aging in quantitative studies, and as advocated by Hale et al. (2022), an intersectional approach is fundamental to understand heterogeneity in cognitive aging inequalities.

### Intersectionality framework

Intersectionality theory emphasizes how multiple social characteristics intersect to create unique social positions with particular exposures to oppression and privilege (Crenshaw, 1990). Analyzing how several social determinants interact and act simultaneously to shape health outcomes is crucial to gain a more nuanced understanding of healthy aging inequalities. Likewise, it is important to understand late-life by situating it within the socially constructed nature of life-course, where privileges and resources are unequally distributed (Holman & Walker, 2021). Continuous exposure to social determinants that dynamically interact throughout the life-course generate cumulative (dis)advantages and greater disparities in late-life cognition (Crimmins, 2020). Thus, the social context is required to fully comprehend the healthy aging process, by considering both accumulation of (dis)advantages over time and through multiple intersecting social determinants. Regarding how social position relates to grandparenthood effects on cognitive functioning, neither studies on grandparental childcare (Ahn & Choi, 2019; Arpino & Bordone, 2014; Sneed & Schulz, 2019; Xu, 2022) nor the only study assessing the transition to grandparenthood (Leimer & van Ewijk, 2022) adopted an intersectional perspective.

### The present study: A longitudinal, intersectional study of grandparenthood and cognitive functioning using MAIHDA

Recent literature in health disparities research advocated for a novel quantitative intersectional methodology, which materialized in the development of Multilevel Analysis of Individual Heterogeneity and Discriminatory Accuracy (MAIHDA) by Clare Evans et al. (2018). MAIHDA is an analytical approach useful to understand how multiple dimensions of social inequality influence health outcomes across and within intersectional strata. Its methodological advantages relate to improved scalability, model parsimony and ability to deal with small subgroup samples (Merlo, 2018). An essential characteristic of MAIHDA is the capability to provide precision-weighted estimates for each intersectional stratum, while overcoming the problem of multiple testing by applying a shrinkage factor to obtain residuals (Bell et al., 2019). An increasing number of studies have applied this framework to investigate how the intersection of (dis)advantaged social identities affect multiple health outcomes (Axelsson Fisk et al., 2018; Bell et al., 2019; Moreno-Agostino et al., 2023). To the best of our knowledge, there has only been one MAIHDA study on biomarkers of healthy aging for older English adults (Holman et al., 2020), therefore more research on healthy aging inequalities with MAIHDA is needed.

The present study aims to shed light on intersectionality, grandparenthood and healthy aging in several ways. Building on previous work that investigated the transition to grandparenthood with data from the Survey of Health, Aging and Retirement in Europe (SHARE) and multilevel modelling (Leimer & van Ewijk, 2022; Tanskanen et al., 2019; Yang, 2021), we integrate MAIHDA with a multilevel longitudinal framework to investigate the research question: *How does the influence of transition to grandparenthood on cognitive functioning vary across intersectional strata?* Particularly, we aim to: (1) determine if intersecting social inequalities explain the variance in late-life cognitive functioning; (2) measure the influence becoming a grandparent on cognitive functioning; and (3) examine how the impact of transition to grandparenthood on cognitive functioning varies across intersectional strata.

## Methods

### Data and Sample

We used data from the regular Waves 1, 2, 4, 5, 6 and 8 of SHARE, which represents a maximum period of 17 years (2004–2020) for 19 European countries (see Supplementary Table S1). SHARE is an extensive European panel study for respondents aged 50 or older and their co-residential partners, with face-to-face interviews that provide multidisciplinary longitudinal data on health, economic and social factors (Börsch-Supan et al., 2013). We selected 19,953 individuals aged 50 to 85 years with more than two observations and with adult children but no grandchildren at the first observation, meaning they could become grandparents during follow-up waves. Each individual had a different baseline wave depending on the time of their first observation, leading to an unbalanced panel data structure.

### Measures

#### Outcome: cognitive functioning

Cognitive functioning was assessed with a single index derived from four validated tests: two tests of memory, one of verbal fluency and one of numeracy. The memory tests involved presenting a list of 10 words to the participants and measuring their immediate recall, and subsequently measuring their delayed recall five minutes later. Verbal fluency was measured by asking the respondents to name as many different animals as they could in one minute. The numeracy test comprised basic arithmetical calculations like subtraction or percentage, based on everyday life situations. We then used Principal Component Analysis (PCA) to summarize the four tests in a unique cognitive measurement for each wave. PCA resulted in only one component explaining more than 60% of the total variance and with Kaiser–Meyer–Olkin (KMO) tests above the 0.70 threshold, hence confirming the adequacy to consider a single index for cognitive functioning (Mazzonna & Peracchi, 2017). Higher scores indicated higher levels of cognitive functioning.

#### Intersectional Strata dimensions

We selected four socio-demographic variables to create unique social positions (i.e., intersectional strata). *Sex/gender* was categorized as women or men, as these were the only categories self-reported in SHARE. Although this binary categorization is not ideal, we used the term sex/gender to account for the conflation of sex and gender in the survey item. *Migration background* was categorized as a binary variable (yes/no), derived from the question “Were you born in the country of interview?” *Education* was classified into low, medium or high education, according to ISCED 1997. *Occupation* was the self-reported present or latest-held work positions, categorized in four groups according to ISCO-88: white-collar high-skill (WCHS), white-collar low-skill (WCLS), blue-collar high-skill (BCHS), and blue-collar low-skill (BCLS). Adults over 50 years are less likely to have occupational change perspectives (Mazzonna & Peracchi, 2017). Given the longitudinal nature of the analysis, we chose four variables that remained unchanged across all waves, and thus positioned individuals in the same intersectional strata over time. The combination of all possible categories resulted in 48 unique intersectional strata based on: sex/gender (2 categories), migration background (2 categories), education (3 categories) and occupation (4 categories) (Evans et al., 2018).

#### Transition to grandparenthood

Transition to grandparenthood was the independent variable. It was captured with a binary variable indicating whether the respondent reported having at least one grandchild at each wave (yes/no) (Sheppard & Monden, 2019; Yang, 2021). To address the limited number of respondents observed throughout the entire study period, we truncated the data at four waves before and after the event (transition to grandparenthood) for analysis. We used this variable as a separate predictor rather than including it as a dimension in the intersectional strata, because it does not contribute to social stratification directly.

#### Covariates

Because cognitive functioning is curvilinear over the life-course (Hale et al., 2022), we adjusted for mean-centered age and a quadratic age term. We also adjusted for a variable of time centered at the transition (*t* = 0 for the first wave when respondents reported having grandchildren). We assigned *t* = 0 randomly for non-grandparent individuals to create an artificial baseline from which to compared those who transitioned to grandparenthood (Sheppard & Monden, 2019).

### Statistical Analyses

MAIHDA analyses are based on fitting multilevel models where individuals are nested within intersectional strata (Evans et al., 2018). In our case, time-varying observations were placed at level 1, nested within individuals at level 2, who were nested within intersectional strata at level 3. We used Restricted Maximum Likelihood (REML) estimation to fit linear multilevel models of the following general form:

$$Y_{ijk}=\beta_{0}+\beta_{1}x_{jk}+\beta_{2}z_{ijk}+v_{0k}+\mu_{0jk}+\epsilon_{0ijk}$$

1 $$\text{Level}\;3:v_{0k}∼N\left(0,\sigma_{v}^{2}\right)\;\text{Level}\;2:\mu_{0jk}∼N\left(0,\sigma_{\mu }^{2}\right)\;\text{Level}\;1:\epsilon_{0ijk}∼N\left(0,\sigma_{\epsilon }^{2}\right)$$

where $Y_{ijk}$ is the cognitive functioning of observation i for individual j in intersectional stratum $k,\beta_{0}$ is the intercept, $x_{jk}$ is a vector of stratum-defining variables, $\beta_{1}$ is the vector with the corresponding parameter values, $z_{ijk}$ is a vector of the observation-level variables including transition to grandparenthood, and $\beta_{2}$ is the vector with the corresponding parameter values. The random effects are captured by the residual variances $\sigma_{v}^{2}$ at the strata-level, $\sigma_{\mu }^{2}$ at the individual-level, and $\sigma_{\epsilon }^{2}$ at the observation-level, and they are all assumed to be uncorrelated.

We first fitted an unadjusted null model (Model 1) to decompose the variance and calculate the Variance Partition Coefficient (VPC). This measure captured the percentage of total variance in the outcome attributable to differences between intersectional strata (Axelsson Fisk et al., 2018). The strata-level VPC was calculated as follows:

2 $$VPC=\frac{\sigma_{v}^{2}}{\sigma_{v}^{2}+\sigma_{\mu }^{2}+\sigma_{\epsilon }^{2}}$$

We added the stratum-defining variables as main effects in Model 2. To quantify the proportion of between-strata variance accounted by the *additive* main effects, we calculated the Proportional Change in Variance (PCV). In case of not explaining the total strata variance (i.e., PCV < 100%), the remaining between-strata variance would indicate the presence of *multiplicative* interaction effects between the intersectional dimensions (Axelsson Fisk et al., 2018). The PCV was calculated as:

3 $$PCV=\frac{\sigma_{v,Nullmodel}^{2}-\sigma_{v,Maineffectsmodel}^{2}}{\sigma_{v,Nullmodel}^{2}}$$

In Model 3 we incorporated covariates as fixed effects to explore the degree to which remaining variance (inequalities) in the outcome could be explained by other factors. By adding the explanatory variable transition to grandparenthood, we accounted for differences in the predicted cognitive functioning between respondents who did or did not become grandparents. The stratum-level residuals (*v*_0*k*_) obtained in Model 3 captured the difference between the stratum-specific means and the value expected based on the *additive* effects. Accordingly, we used the residuals to isolate the *multiplicative* effects that show the intersectional interaction effects in each stratum.

We expanded MAIHDA analyses with multilevel longitudinal models in a stepwise procedure, evaluating the adequacy of including random slopes by comparing the fit of each subsequent model with a likelihood ratio test (LRT) (LaHuis & Ferguson, 2009). First, we fitted Model 4 by adding a random slope on transition to grandparenthood (Heisig & Schaeffer, 2019). The aim of this model was to capture possible intersectional variation in the effect of becoming a grandparent. Subsequently, in Model 5 we added cross-level interactions of sex/gender, migration background, education and occupation with the transition to grandparenthood. The expanded Model 5 took the form:

4 $$Y_{ijk}=\beta_{0}+\beta_{1}x_{jk}+\beta_{2}z_{ijk}+\beta_{3}x_{jk}Grand_{ijk}+v_{k}+\mu_{0jk}+\epsilon_{0ijk}$$

5 $$v_{k}=v_{0k}+v_{1k}Grand_{ijk}$$

where notation is equivalent to (1) with the addition of the cross-level interactions composed by $Grand_{ijk}$ representing the transition to grandparenthood at the observation-level, $x_{jk}$ representing a vector of the strata-defining variables, and $\beta_{3}$ a vector with the associated parameter values. Stratum residuals were composed by a random intercept, namely $v_{0k}$, and a random slope $v_{1k}$ on the transition to grandparenthood. This model depicted how much variation in the random slope remained after accounting for the additive effects of the strata-defining variables, but in this case for the longitudinal association between grandparenthood and cognitive functioning rather than for the overall level of the latter.

We conducted a comparison of the random slope variance between Model 4 and Model 5 to measure if some variance remained after adding the cross-level interactions. This would suggest that intersectional effects could explain the remaining variance in cognitive functioning trajectories. Finally, we tested the adequacy of adding random slopes by comparing the fit of Model 5 and Model 6, which incorporated the cross-level interactions but omitted the random slope. As our approach extended the conventional cross-sectional MAIHDA, the VPC and PCV became conditional to the added random slopes, hence the straight-forward interpretation was diminished and prevented us from direct general comparisons to the null model (Goldstein et al., 2002). All analyses were conducted in Stata 17.0 considering a two-tailed p-value < 0.05 for statistical significance.

## Results

### Descriptive Statistics

Table 1 presents the descriptive statistics of the study population at *t* = −1, differentiated by whether the respondents experienced the transition to grandparenthood or not. The sample consisted of 19,953 individuals and 62,386 observations, with an average of 3.13 observations per respondent. Overall, the distribution of the strata-defining social determinants was remarkably similar between individuals who became grandparents (46.50%) and those who did not (53.50%). Our analysis included a slightly higher proportion of women (52.21%). The sample was diverse enough, with about 10% of respondents reporting a migration background. More than 40% had medium education, while almost a third were highly educated. In respect to occupation, most respondents reported either WCHS (37.60%) or WCLS (32.33%), followed by BCHS (15.14%) and BCLS (14.92%). Individuals who became grandparents were slightly older at baseline (61.71 years) compared to those who did not (60.98 years). Finally, the sample mean cognitive functioning was very similar for grandparents (27.08) and non-grandparents (26.80).

### MAIHDA I: Intersectional variation in the level of cognitive functioning

Results obtained from all MAIHDA models are presented in Table 2. The VPC in Model 1 indicated that 17.43% of the total variance in cognitive functioning was attributable to the intersectional strata, revealing a good level of clustering (Axelsson Fisk et al., 2018). This suggested that the intersectional strata played a substantial role in explaining cognitive functioning inequalities. When adding the dimensions of intersectional strata as main effects in Model 2, the VPC was reduced to 0.76% and the PCV was 96.39%. Model 3 showed better fit with a significant LRT (χ^2^ = 2775.6; p < 0.01), demonstrating that random effects were jointly significant. Including covariates in this model further reduced the VPC (0.51%) and increased the PCV (97.71%), leaving only an unexplained strata-level variance of 2.29% (100% - PCV). This implied that most of the differences in cognitive functioning across intersectional strata were due to additive effects of sex/gender, migration background, education and occupation.

**Figure 1:**
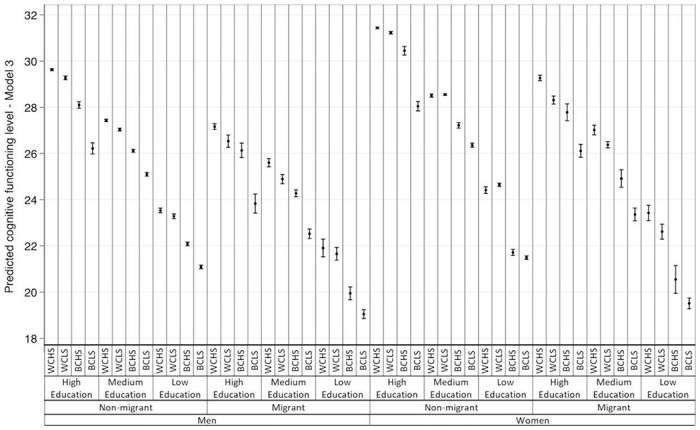
Predicted cognitive functioning level with 95% confidence intervals, by intersectional strata (Model 3) WCHS: White-Collar High-Skill; WCLS: White-Collar Low-Skill; BCHS: Blue-Collar High-Skill; BCLS: Blue-Collar Low-Skill.

**Figure 2:**
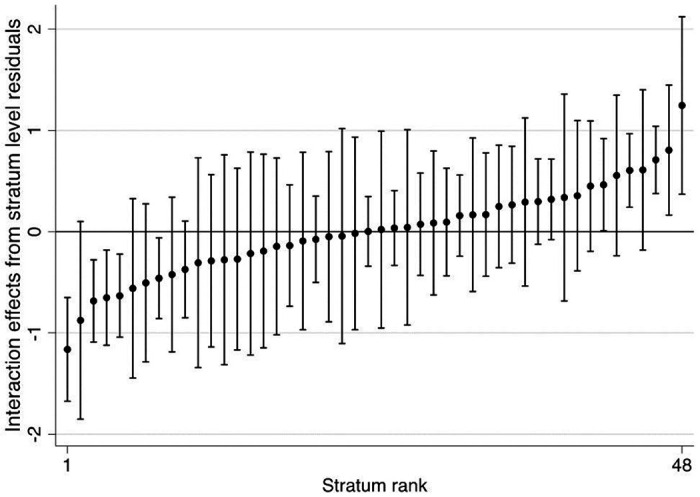
Stratum level residuals with 95% confidence intervals from Model 3. Intersectional strata are presented in ascendant ranking of their residual values. Each stratum is labelled with a four-digit code corresponding to the social strata dimensions in the following order: Sex/gender: 1= Men, 2 = Women; Migration Background: 1 = No, 2 = Yes; Education: 1 = High, 2 = Medium, 3 = Low; Occupation: 1 = White-Collar High-Skill, 2 = White-Collar Low-Skill, 3 = Blue-Collar High-Skill, 4 = Blue-Collar Low-Skill.

Figure 1 illustrates the heterogeneity in predicted cognitive functioning between intersectional strata based on Model 3, considering the total effects (additive and multiplicative effects). Figure 2 shows the stratum-level residuals from Model 3 (multiplicative effects), which represent the intersectional interaction effects. Only five strata had significantly higher cognitive functioning than expected from the additive main effects only (CIs not including 0), whereas another five strata had significantly lower cognitive functioning than expected. Table 3 contains more detailed information on the residual analysis, displaying the five intersectional strata with highest and lowest interaction effects. Although we found some modest intersectional effects, these results demonstrated that the differences between strata were mostly driven by additive effects, consistent with the low VPC and high PCV observed in Model 3. This indicated that the multiplicative effects were small.

Regarding the transition to grandparenthood, Model 3 revealed that individuals who became grandparents had a higher cognitive functioning than those who did not become grandparents (coefficient = 0.79, p < 0.01). These differences were averaged over all intersectional strata, and consistently present in longitudinal Models 4–6 (coefficients ranging from 0.79 to 0.75, p < 0.01, see Supplementary Table S4).

### MAIHDA II: Intersectional variation in the effect of grandparenthood on cognitive functioning trajectories

Regarding the longitudinal MAIHDA in Table 2, a significant LRT (χ^2^ = 8.03; p = 0.02) confirmed the better fit of Model 4 compared to Model 3, hence allowing the slope of transition to grandparenthood to vary across strata. Model 5 had a better fit than Model 4, with a significantly LRT (χ^2^ = 5.11; p = 0.03). Our findings indicated that there was some remaining random slope variance after adding cross-level interactions, suggesting that the impact of transitioning to grandparenthood on trajectories of cognitive functioning had certain variability across intersectional strata. There was one significant interaction between transition to grandparenthood and sex/gender (coefficient = 0.31, p = 0.03), indicating that becoming a grandmother had multiplicatively positive effects on cognitive functioning for women. The comparison between Model 6 and Model 5 resulted in a worse fit of the former, with a non-significant LRT (χ^2^ = 3.78; p = 0.15). This revealed that a random slope for the transition to grandparenthood was adequate (see Supplementary Table S4). Since only one of the interactions with the strata-defining variables was significant, we did not find clear evidence that intersectional strata influenced the link between the transition to grandparenthood and cognitive functioning.

## Discussion

Our main aim was to investigate how intersectional social positions and the transition to grandparenthood are associated with late-life cognitive functioning in a European population. Using SHARE longitudinal data, we applied MAIHDA to explore heterogeneities in social determinants of cognitive functioning with an intersectional lens. Moreover, we expanded this methodology in a longitudinal manner to capture changes in cognitive functioning trajectories after the transition to grandparenthood. Our findings showed clear cognitive functioning differences across social positions measured at the intersection of sex/gender, migration background, education and occupation. Most of these differences were explained by additive rather than multiplicative effects, hence finding only modest intersectional interaction effects. The transition to grandparenthood was associated with a higher level of late-life cognitive functioning. Yet, we found no clear evidence of intersectional effects in the association between transition to grandparenthood and cognitive functioning. Results suggested that women who became grandmothers had a larger benefit in their cognitive functioning trajectories than men or women who did not become grandmothers. While becoming a grandparent is an important transition associated with higher levels of cognitive functioning, the role of social position in shaping cognitive functioning and health inequalities cannot be overlooked.

There were substantial inequalities in cognitive functioning across intersectional strata, and most importantly, these heterogeneities showed a clear social gradient. Respondents with combinations of disadvantaged social determinants, such as migration background, low education and blue-collar occupations exhibited lower cognitive functioning. These results are aligned with the scarce literature on intersectionality of cognitive inequalities, which highlights that cognitive decline in late-life is of special concern for populations experiencing multiple forms of social inequalities (Hale et al., 2022; Walsemann et al., 2022). We found limited evidence of multiplicative effects. Nevertheless, the uncovering of meaningful variation across intersectional strata should be seen as a step further in unraveling social heterogeneities, emphasizing the importance of exploring intersectional mechanisms to gain a more nuanced understanding of disparities in healthy aging.

Our results suggested that the transition to grandparenthood may contribute to successful aging by increasing overall levels of cognitive functioning or delaying its decline. This broadens previous work that showed some cognitive benefit associated with grandparenting (Ahn & Choi, 2019; Arpino & Bordone, 2014; Bordone & Arpino, 2019; Sneed & Schulz, 2019; Xu, 2022) or becoming a grandparent (Leimer & van Ewijk, 2022), especially for women. Grandparenthood is often associated to increased social and emotional connections with the younger generations and stronger intergenerational ties, which may be particularly beneficial for cognitive functioning (Krzeczkowska et al., 2021). Hence, our findings are aligned with the cognitive enrichment theory (Hertzog et al., 2008), suggesting that the role acquired and feelings derived from intergenerational ties promote intellectual stimulation that help maintain cognitive functioning or even prevent its decline.

We did not find clear evidence about how intersectional strata explain the heterogeneity in the impact of the transition to grandparenthood on cognitive functioning. Nonetheless, we found that becoming a grandmother can be particularly beneficial for women. Similar to Sheppard and Monden (2019) and Di Gessa et al. (2020), we argue that these sex/gender differences are aligned with the kin-keeper argument, as women traditionally devote more time and effort in maintaining intergenerational relationships. While there is evidence that women provide more grandchild care and this may imply cognitive functioning benefits (Arpino & Bordone, 2014), the adoption of the role itself similarly contributes by strengthening grandmothers’ binding positions within intergenerational ties (Sieber, 1974).

MAIHDA is proved as a useful tool to map health inequalities in aging societies and accurately design precision public health actions. However, there is an ongoing debate about the inference of level-2 residuals as significant interactions in multilevel models, since it is subject to an increased chance of erroneously detecting interactions due to multiple testing; some scholars advocate to control the overall error rate by inflating the confidence intervals with corrections such as Bonferroni (Afshartous & Wolf, 2007); alternatively, others argue that multilevel models address this problem with the shrinkage that automatically occurs when obtaining residual estimates (Bell et al., 2019; Jones et al., 2016). As demonstrated by Gelman et al. (2012), the shrinkage may be more efficient as it leads to more appropriate conservative comparisons without reducing the power to detect true differences. While acknowledging this ambiguity, we opted to rely on the shrinkage to estimate stratum-level residuals, instead of applying multiple-testing corrections. Since our results show low stratum-level variation after adding the main effects, we are likely to be in a high shrinkage and therefore conservative inference setting (Bell et al., 2019).

This study has several strengths. The sample was based on data from a large, multi-national longitudinal survey. We adopted a specific application of quantitative intersectionality by mapping cognitive functioning inequalities across intersectional strata, which, to our knowledge, has not been yet investigated. Further, we analyzed the longitudinal associations between the transition to grandparenthood and cognitive functioning, finding remarkable benefits of intergenerational ties for healthy aging. Additionally, we extended MAIHDA with a longitudinal application, an important step for future research on intersectional trajectories.

### Limitations and Future Directions

Various limitations need to be considered. First, SHARE does not provide the exact date of grandchild birth thus we could only assume a date between the two reporting periods to estimate the effect. Second, we used an unbalanced longitudinal sample where a majority of observations were pooled around the event of study. While a balanced longitudinal dataset would allow for better control of trajectories in the outcome, we opted to favor a larger sample size. Third, our sample could be subject to selective panel attrition since older adults with particularly higher levels of cognitive functioning and well-being are more likely to participate in follow-up waves. Fourth, we only examined short-term effects of grandchild birth, it is possible that we were not capturing the long-term association with cognitive functioning.

Our study makes an important contribution by indicating the importance of adopting an intersectional lens to understand later life and cumulative (dis)advantages of an increasingly heterogeneous population. We provide evidence about the preventive advantages that intergenerational relationships add to the healthy aging of grandparents. Accordingly, precision public health interventions should be oriented towards fostering the intergenerational chain with an intersectional focus.

## Conclusion

Our results revealed substantial cognitive functioning differences across intersectional social positions. These differences were mostly due to additive effects, underlining the important role of social determinants for trajectories of cognitive functioning across the life course. Further, we found evidence that the transition to grandparenthood is positively associated with late-life cognitive functioning. As societies grow older and more people become grandparents, this is paramount for understanding the process of healthy aging. Fostering intergenerational exchange while considering social determinants and intersectionality holds potential as a strategy for preserving late-life cognitive functioning.

## Figures and Tables

**Table 1:** Descriptive sample statistics at baseline (*t* = −1), N = 19,953.

Variables	Transition to grandparenthood	No transition to grandparenthood	Total
	%	%	N
**Dimensions of social position**			
Sex			
Men	46.70	48.73	9,535
Women	53.30	51.27	10,418
Migration background			
No migration background	89.98	89.03	17,853
Migration background	10.02	10.97	2,100
Education			
High	29.25	33.02	6,238
Medium	42.80	40.33	8,276
Low	27.95	26.65	5,438
Occupation			
White-collar high-skill (WCHS)	38.01	37.24	7,503
White-collar low-skill (WCLS)	32.77	31.95	6,451
Blue-collar high-skill (BCHS)	14.67	15.56	3,022
Blue-collar low-skill (BCLS)	14.55	15.25	2,978
**Covariates**			
Age, M (SD)	61.71 (7.10)	60.98 (7.41)	61.35 (7.27)
**Outcome**			
Cognitive functioning, M (SD)	27.08 (7.50)	26.80 (7.64)	26.95 (7.57)
Total individuals	9,279	10,674	19,953

*Note: t* = time; M = mean; SD = standard deviation. Unweighted estimates.

**Table 2:** Results from MAIHDA intersectional models for level of cognitive functioning.

	Model 1 (Null)	Model 2 (Main effects)	Model 3 (Adjusted)	Model 4 (Random Slope)	Model 5 (Interactions)
Fixed Effects	Coefficient (95% CI)	Coefficient (95% CI)	Coefficient (95% CI)	Coefficient (95% CI)	Coefficient (95% CI)
Intercept	25.34* (24.44, 26.25)	29.75* (29.18, 30.32)	30.60* (30.12, 31.09)	30.60* (30.16, 31.03)	30.59* (30.15, 31.02)
*Intersectional strata*					
Men		Ref.	Ref.	Ref.	Ref.
Women		1.30* (0.86, 1.74)	1.10* (0.72, 1.47)	1.04* (0.70, 1.39)	1.05* (0.71, 1.4)
No migration background		Ref.	Ref.	Ref.	Ref.
Migration background		−2.12* (−2.59, −1.66)	−2.07* (−2.47, −1.66)	−2.07* (−2.46, −1.68)	−2.08* (−2.47, −1.69)
High education		Ref.	Ref.	Ref.	Ref.
Medium education		−2.07* (−2.62, −1.53)	−2.03* (−2.49, −1.57)	−2.03* (−2.46, −1.60)	−2.04* (−2.47, −1.61)
Low education		−6.16* (−6.73, −5.59)	−5.72* (−6.21, −5.24)	−5.71* (−6.17, −5.25)	−5.72* (−6.17, −5.26)
White-collar High skill		Ref.	Ref.	Ref.	Ref.
White-collar Low-skill		−0.41 (−0.99, 0.18)	−0.58* (−1.07, −0.09)	−0.54* (−0.99, −0.09)	−0.52* (−0.95, −0.08)
Blue-collar High-skill		−1.64* (−2.27, −1.01)	−1.76* (−2.30, −1.22)	−1.73* (−2.24, −1.23)	−1.73* (−2.23, −1.23)
Blue-collar Low-skill		−3.08* (−3.72, −2.44)	−3.26* (−3.80, −2.72)	−3.18* (−3.69, −2.67)	−3.18* (−3.68, −2.67)
*Covariates*					
Age			−0.17* (−0.19, −0.16)	−0.17* (−0.19, −0.16)	−0.17* (−0.19, −0.16)
Age quadratic			−0.01* (−0.01, −0.01)	−0.01* (−0.01, −0.01)	−0.01* (−0.01, −0.01)
Time since first grandparenthood			0.45* (0.40, 0.49)	0.45* (0.40, 0.49)	0.43* (0.37, 0.49)
Not grandparent			Ref.	Ref.	Ref.
Grandparent			0.79* (0.67, 0.91)	0.79* (0.66, 0.92)	0.75* (0.49, 1.01)
*Interactions*					
Grandparent*Women					0.31* (0.08, 0.55)
Grandparent*Migration					0.13 (−0.23, 0.50)
Grandparent*Med. education					0.02 (−0.27, 0.31)
Grandparent*Low education					0.05 (−0.28, 0.38)
Grandparent*WCLS					−0.01 (−0.26, 0.27)
Grandparent*BCHS					−0.04 (−0.38, 0.29)
Grandparent*BCLS					−0.19 (−0.53, 0.15)
**Random Effects**					
Between stratum variance	10.046 (6.614, 15.260)	0.363 (0.165, 0.797)	0.231 (0.094, 0.562)	0.190 (0.067, 0.527)	0.151 (0.071, 2.059)
Grandparent (random slope)				0.130 (0.008, 0.209)	0.088 (0.052, 1.978)
VPC (%)	17.43%	0.76%	0.51%	-	-
**PCV (%)**	-	96.39%	97.71%	-	-

*Notes:* CI: Confidence Interval; WCLS: White-Collar Low-Skill; BCHS: Blue-Collar High-Skill; BCLS: Blue-Collar Low-Skill; VPC: Variance Partition Coefficient; PCV: Proportional Change in Variance. Models 3–5 control for country dummies.

* p < .05.

**Table 3.** Five intersectional strata with the highest and lowest residuals (intersectional interaction effects) in Model 3, with 95% confidence intervals.

	Sex/Gender		Migration Background		Education	Occupation	

Stratum	M	W	No	Yes	Hi Me Lo WCHSWCLSBCHSBCLS		Intersectional interaction effects (95% CI)
	Five Strata with the most positive (protective) Interaction Effects						

2113							**1.25 (0.37, 2.12)**
	
2222							**0.81 (0.16, 1.45)**

2122							**0.71 (0.38, 1.04)**

2221							0.61 (−0.18, 1.4)

1334							**0.61 (0.24, 0.97)**

	Five Strata with the most negative (hazardous) Interaction Effects						

1134							**−0.63 (−1.04, −0.22)**

1132							**−0.65 (−1.12, −0.18)**

2134							**−0.68 (−1.09, −0.28)**

1214							−0.88 (−1.85, 0.10)

2133							**−1.16 (−1.67, −0.65)**

*Notes:* M: Men; W: Women; Hi: High; Me: Medium; Lo: Low; WCLS: White-Collar Low-Skill; BCHS: Blue-Collar High-Skill; BCLS: Blue-Collar Low-Skill; CI: Confidence Interval.
